# FT-MDNNMDs: early detection of breast cancer using fine-tuned multi-deep neural networks with TCGA and clinical image datasets

**DOI:** 10.1038/s41598-026-47731-z

**Published:** 2026-04-15

**Authors:** Arslan Shafique, Abid Mehmood, Rabiah Al-Qudah, Khiati Zakaria

**Affiliations:** 1 James Watt School of Engineering, University of Glasgow, Glasgow, G12 8QQ UK; 2 Department of Computer Science, Abu Dhabi University, Abu Dhabi, 59911 UAE

**Keywords:** Cancer, Computational biology and bioinformatics, Mathematics and computing, Oncology

## Abstract

Breast cancer remains one of the leading causes of cancer-related mortality among women worldwide. Early and accurate detection is critical for improving survival rates and enabling effective treatment. This research proposes a fine-tuned Multi-Deep Neural Network model with Multiple Datasets (FT-MDNNMDs). The proposed system employs a hierarchical classification strategy, first distinguishing cancer from normal cases, and subsequently classifying cancer-positive cases into Stage II and Stage III categories. The model integrates data from The Cancer Genome Atlas (TCGA) and a private clinical image dataset collected from hospitals in Pakistan. Preprocessing techniques, including image normalization, filtering, and Principal Component Analysis (PCA), are applied to enhance feature quality and reduce redundancy. Transfer learning and fine-tuning strategies are incorporated to further improve classification performance. The proposed model effectively distinguishes between benign and malignant tumors and accurately identifies stage II and stage III cases. Experimental results demonstrate that the fine-tuned MDNNMDs model achieves an accuracy of 99.57%, outperforming conventional machine learning algorithms such as Support Vector Machine (94.46%), Decision Tree (93.54%), and Naïve Bayes (91.22%). The model also achieved superior MCC (99.46%), F-score (99.57%), and recall (99.63%). In addition to algorithmic comparison, the performance of the proposed model is compared with existing breast cancer detection models, and it is revealed that the proposed model provides more accurate results than existing ones, with 1.5% to 3.0% better accuracy.

## Introduction

Breast cancer is a global health concern and is the second most common cause of cancer-related deaths among women worldwide^1,2^. It arises primarily in the breast lobules or ducts and can progress rapidly if not detected and treated in its earlier stages. According to recent statistics, breast cancer accounts for a significant percentage of new cancer diagnoses annually, and its incidence is increasing due to factors such as urbanisation, lifestyle changes, and lack of access to early screening in many parts of the world. Early and accurate detection of breast cancer is necessary because it can dramatically improve survival rates and treatment effectiveness. In particular, stages II and III of breast cancer represent more critical diagnostic window. At these stages, the tumor has typically begun to spread to nearby tissues or lymph nodes, but has not yet reached distant organs. Timely diagnosis at this point enables more aggressive and targeted treatment options. However, despite their clinical importance, stages II and III are often harder to detect reliably due to the subtlety of visual features and overlapping characteristics with benign tissue, especially in dense breast profiles^3,4^. Moreover, medical imaging techniques such as mammography, ultrasound, and magnetic resonance imaging (MRI) are widely used for breast cancer screening and diagnosis. However, each of these methods has its own limitations, such as reduced sensitivity in dense breast tissue, high false-positive rates, or limited accessibility and cost-effectiveness. Their diagnostic accuracy depends heavily on the expertise of the radiologist and the quality of the imaging equipment. Further, the interpretation of imaging results is often subjective; that leads to variability in diagnosis and the possibility of false positives or negatives^5^. Histopathological confirmation through biopsy is a very high standard, but it is invasive, time-consuming, and potentially associated with procedural risks such as the spread of malignant cells^6^.

**Fig. 1:**
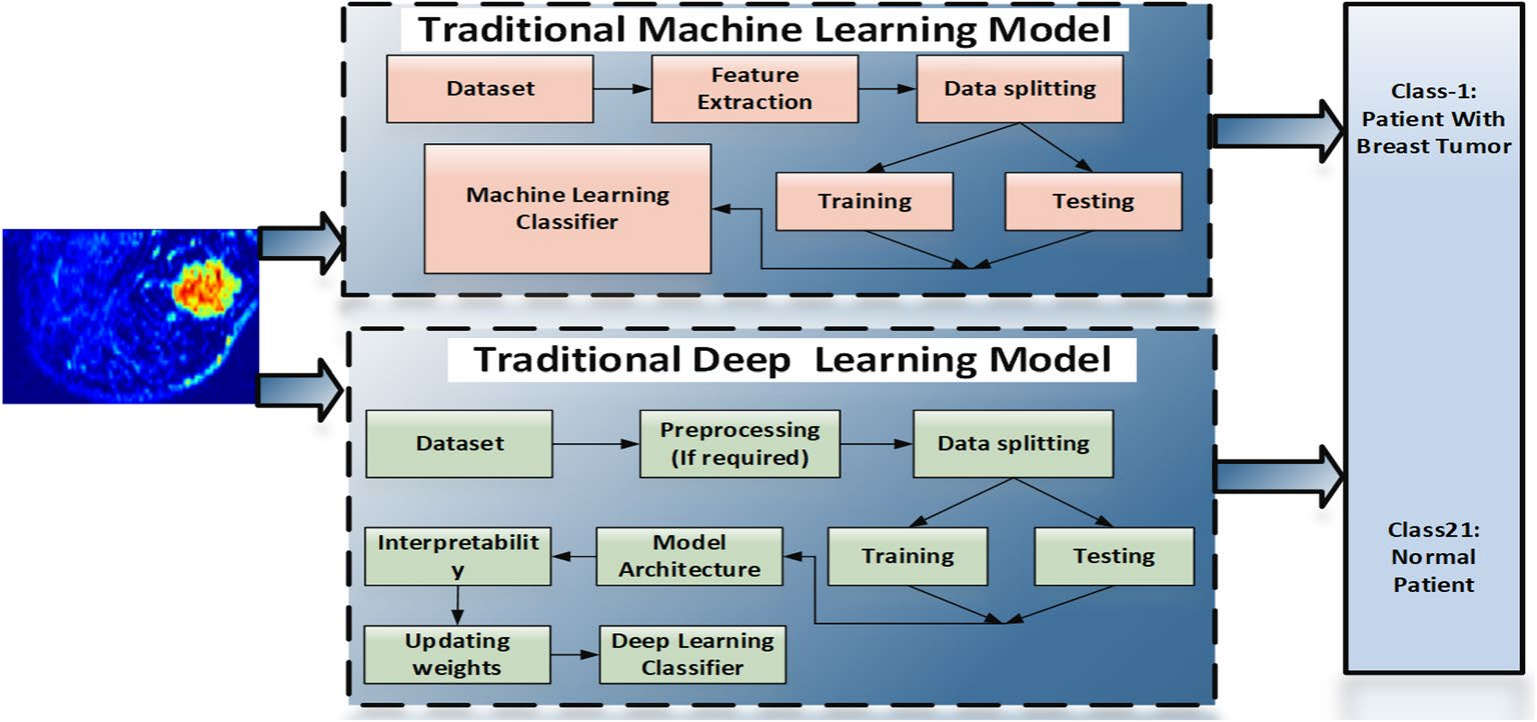
Traditional machine learning and deep learning frameworks.

In response to these limitations, there has been a growing interest in the application of artificial intelligence (AI) and deep learning in medical diagnostics. These techniques have shown substantial promise in automating image analysis, reducing diagnostic errors, and enhancing clinical decision-making. Unlike traditional machine learning models that rely on manually engineered features, deep learning models can automatically learn hierarchical patterns directly from raw image data, making them particularly well-suited for complex classification tasks such as differentiating between benign and malignant lesions. Despite their potential, many existing AI-based breast cancer detection models still face notable challenges. This distinction between traditional machine learning and deep learning approaches is illustrated in Fig. 1.

### Traditional machine learning-based approaches

Over the past few years, numerous researchers have proposed methods leveraging machine learning and deep learning for breast cancer detection. More recently, most breast cancer classification models have been developed using conventional machine learning (ML) techniques based on two well-established imaging modalities, often referred to as “gold standards”: grayscale mammography and colored hyperspectral imaging (HI)^7–11^. For example, Kowal et al.^12^ implemented nuclear segmentation using techniques such as fuzzy C-means, Gaussian mixture^13^, and competitive neural network models^14^ to validate segmentation results. The experiments involved 600 microscopic biopsy images from 60 patients. Additionally, 45 morphological, texture, and topological features were extracted from the segmented region of interest (ROI) to generate a Morphological Feature Vector (MFV). Subsequently, NB, and DT were applied to MFV to develop a model for classification task that produce an accuracy of 96z

In^15^, Zemouri et al. developed a computer-aided breast cancer diagnosis (BC-CAD) system that uses a deep structural neural network to predict the Recurrence Score (RS) of the Oncotype DX (ODX) assay. The ConstDeepNet algorithm created two classifiers, employing a one-versus-all structure in the first architecture and using one Deep Neural Network for all three classes in the second architecture. In^16^, Nabawy et al. proposed a model for the classification of breast cancer that uses feature fusion in METABRIC datasets. Employing various machine learning classifiers, including SVM, Random Forests (RF), and Boosting, 88.36% accuracy was achieved. In^17^, Zhang et al, proposed an unsupervised feature learning framework that integrates principal component analysis (PCA) and neural network to extract diverse features. The obtained features were utilized to construct an ensemble classifier based on the AdaBoost algorithm for predicting clinical outcomes in breast cancer. The comparative experiments included a baseline classifier (PCA-Ada) with the same learning strategy, but differing in training inputs. Arravalli et al.^18^ explored machine learning classifiers with explainable AI techniques for breast cancer prediction using diagnostic patient features. Random Forest achieved the best F1-score (84%), and interpretability methods such as SHAP and LIME enhanced model transparency for clinical decision support.

### Deep learning-based imaging approaches

A few existing studies explored advanced deep learning frameworks for cancer detection across various imaging modalities. For instance, yemeesha et al.,^19^ proposed an unmanned transfer learning-based probabilistic multi-layer dense network for skin cancer detection, demonstrating the effectiveness of transfer learning in dermatological image classification. Similarly, Kumar et al.,^20^ combined Taylor-Bird Squirrel Optimization with a deep recurrent neural network and applied for prostate cancer classification using MRI data to highlight the importance of optimization strategies and deep feature extraction in medical imaging analysis. Arshad et al.^21^ propose HistoDX, an EfficientNetV2-B3-based framework for IDC classification, achieving 97% accuracy and strong generalizability across BreakHis and BACH datasets. Their work effectively addresses class imbalance and demonstrates clinical potential, though challenges remain in detecting underrepresented IDC(+) samples. Khushi et al.^22^ present an EfficientNetB4-based model for brain tumor classification, achieving up to 99.87% accuracy with extensive augmentation and optimized training strategies. The study reports exceptional performance metrics, but its reliance on augmented data may limit real-world generalization and robustness. Arshad et al.^21^ proposed HistoDX, an EfficientNetV2-B3-based deep learning framework for invasive ductal carcinoma (IDC) classification using large-scale histopathology image patches. The model achieved 97% accuracy and demonstrated cross-dataset generalizability on BreakHis and BACH, though detection of minority IDC(+) cases remained challenging. Gardezi et al.^23^ provided a comprehensive overview of machine learning advancements in medical imaging, highlighting the transition from handcrafted feature-based CAD systems to deep learning-driven automated diagnostic frameworks. Their work emphasizes the growing impact of hierarchical feature learning in enhancing breast cancer detection accuracy. Saharan et al.^24^ introduced “DXAIB,” a hybrid CNN–Random Forest model integrated with SHAP-based explainability to enhance transparency in breast cancer detection. The framework improves diagnostic accuracy while addressing interpretability concerns critical for clinical trust. Vijayalakshmi et al.^25^ developed a hybrid classification approach combining handcrafted texture features and deep learning (BiLSTM-CNN) for mammogram analysis. Evaluated on the MIAS dataset, the method achieved 97.14% accuracy, demonstrating the effectiveness of combining statistical descriptors with deep models.

### Dataset development and multimodal extensions

The two studies mentioned above demonstrated improved performance in breast cancer image classification. However, these studies face two significant limitations. Firstly, they utilized a dataset with a limited number of training images, and also, these images are taken from a single dataset. Consequently, the models proposed in these studies may lack generalizability and cannot be easily applied on a larger scale. Second, these studies focused on binary classification problems, categorizing images as benign or malignant. Yet, breast cancer images can be more precisely classified into various specific types. To overcome these challenges, Spanhol et al.^26^ introduced a substantial dataset called BreakHis. This dataset comprises 7080 images from 85 patients. Furthermore, breast cancer images within this dataset are classified into nine different types of breast cancer. Brahmareddy et al.^27^ proposed TransBreastNet, a CNN–Transformer hybrid architecture that jointly models spatial, temporal, and clinical metadata for breast cancer subtype and stage prediction. Their multimodal multitask framework achieved strong performance on mammogram datasets and incorporated built-in explainability modules.

The summary of the existing work is given in Table 1.

**Table 1:** Summary of representative breast cancer detection studies.

Study	Method type	Dataset	Task	Key limitation
Kowal et al.^12^	ML (NB, DT)	Biopsy images	Binary	Small dataset
Hu et al.^28^	ML + Segmentation	ROI images	Binary	Limited generalization
Tripathy et al.^29^	AI texture features	Cellular images	Binary	Single modality
Zemouri et al.^15^	Deep structural NN	ODX data	Multi-class	No imaging data
Cruz et al.^30^	CNN	TCGA histology	Tumor detection	Single modality
Murtaza et al.^31^	CNN (BMIC-Net)	BreakHis	Multi-class	Dataset specific
Nabawy et al.^16^	ML fusion	METABRIC	Outcome prediction	No imaging
Zhang et al.^17^	PCA + NN	Clinical data	Outcome prediction	Limited fusion
Nyemeesha et al.^19^	Transfer learning	Skin images	Binary	Cancer specific
Kumar et al.^20^	DRNN + Optimization	MRI prostate	Binary	Modality specific
Arshad et al.^21^	EfficientNetV2-B3	BreakHis/BACH	IDC classification	Class imbalance
Khushi et al.^22^	EfficientNetB4	Brain MRI	Multi-class	Heavy augmentation
Gardezi et al.^23^	ML/DL review	Multiple datasets	Survey	No experiments
Saharan et al.^24^	CNN + RF	Breast images	Binary	Limited integration
Brahmareddy et al.^27^	CNN-Transformer	Mammogram + metadata	Subtype + stage	No genomics
Vijayalakshmi et al.^25^	BiLSTM-CNN	MIAS	Binary	Dataset specific
Arravalli et al.^18^	ML + XAI	Clinical features	Binary	Moderate accuracy
Proposed FT-MDNNMDs	Multimodal FT-DNN	Imaging + genomics	Stage-level	Integrated framework

Despite significant advances in deep learning for breast cancer detection, several limitations remain in existing early-stage diagnostic approaches. Many existing studies rely on single-modality imaging data, such as mammography or histopathology alone, which may not fully capture the heterogeneous nature of tumor characteristics. Additionally, limited integration of structured clinical and genomic information restricts the ability of models to leverage complementary diagnostic signals. Several existing frameworks focus primarily on binary cancer detection (cancer vs. normal) without performing detailed stage-level stratification, which is clinically important for treatment planning. Furthermore, many reported models are validated on isolated datasets without addressing cross-dataset heterogeneity or multimodal fusion challenges. In view of the existing challenges, this research proposes a deep learning-based diagnostic framework with a targeted focus on breast cancer stages II and III. The key novelty of the proposed MDNNMDs framework lies in its ability to integrate heterogeneous data sources, including multimodal imaging, structured clinical parameters, and genomic features, within a unified architecture. Unlike conventional deep learning models that focus on single-modality imaging datasets, the proposed approach further incorporates transfer learning, fine-tuning strategies, and weighted score-level fusion to enhance robustness and stage-level discrimination. This novelty in multimodal integration and stage-aware modeling clearly differentiates MDNNMDs from existing deep learning-based breast cancer detection systems.

- Unlike conventional models that treat breast cancer detection as a general classification task, the proposed research introduces a framework that is explicitly designed to identify and differentiate between stages II and III, which are critical for timely clinical intervention.
- The proposed model incorporates both publicly available datasets and a private clinical dataset collected from several hospitals. This multi-source data integration enhances generalizability and makes the system adaptable to diverse imaging conditions and population variations.
- Leveraging deep learning’s ability to perform hierarchical feature extraction, the proposed system can detect subtle differences in tumor morphology and texture without relying on manual or handcrafted features.
- The proposed approach is designed to handle real-world clinical noise and image quality inconsistencies, which are very common challenges in breast cancer imaging, especially in low-resource settings.
- By focusing on the early identification of stages II and III, the proposed model directly supports clinical decision-making which enable the earlier treatment initiation and potentially reduces overall breast cancer mortality rates.

### Problem formulation

To clarify the research objectives, the proposed framework addresses breast cancer detection using a hierarchical classification strategy. The primary task is binary classification to distinguish cancer cases from normal patients. Subsequently, for the cancer-positive cases, a secondary classification task is performed to differentiate between Stage II and Stage III breast cancer. Within these stages, tumors are further categorized as benign or malignant based on clinical and imaging characteristics. Therefore, the overall prediction framework combines binary and stage-level classification to support early clinical decision-making for mid-stage breast cancer detection.

### Paper structure

The remainder of the paper is organized as follows: “Material and methods” section describes the materials and methods used to develop the proposed AI-based early breast cancer detection system. “Experimental results, analysis, and discussion” section presents the experimental results and performance analysis of the proposed model, along with a comparison to existing approaches. “Limitations and deployment challenges” section provides the limitations and future deployment challenges. Finally, “Conclusion” section concludes the paper by summarising the key findings.

## Material and methods

**Fig. 2:**
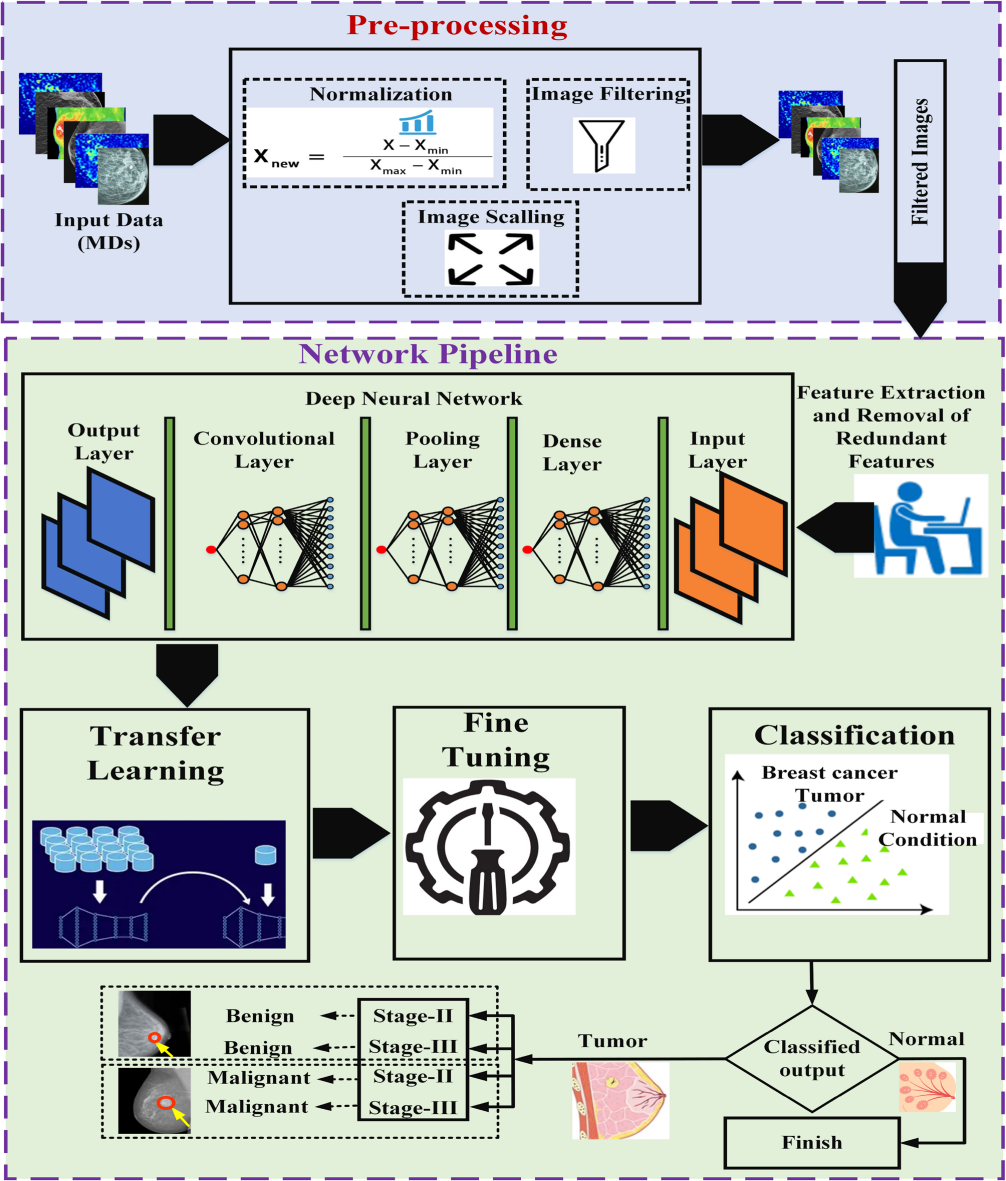
Early breast cancer tumor detection using the proposed MDNNMDs.

This research proposes a deep learning method utilizing multi-deep neural networks for the early detection of breast cancer, particularly targeting stages II and III. Achieving high precision with CNN necessitates a large training dataset. However, commonly used datasets such as TCGA has limited instances. Relying on a single dataset may lead to decreased accuracy in final classifications. To address this limitation, a publicly available dataset is integrated with the TCGA dataset to expand the dataset. The flow of the proposed early breast cancer detection model is depicted in Fig. 2.

### Definition of classification tasks

To ensure clarity in problem formulation, the proposed framework addresses three related classification tasks:

- Task 1: Normal vs. Tumor Classification: This primary screening task distinguishes normal patients from patients diagnosed with breast tumors.
- Task 2: Benign vs. Malignant Classification: For tumor-positive cases, the model differentiates between benign and malignant tumors based on imaging and clinical features.
- Task 3: Stage II vs. Stage III Classification: For malignant tumor cases, the model further performs stage-level classification to distinguish between Stage II and Stage III breast cancer.

All reported performance metrics in the “Experimental results, analysis, and discussion” section are explicitly associated with these tasks to ensure transparency and interpretability.

#### TCGA cohort selection and data modalities

In this research, the TCGA-BRCA cohort is used from The Cancer Genome Atlas (TCGA). A total of N_TCGA_ patients/tumor cases are included after applying the following inclusion criteria:

1. Availability of required clinical variables (e.g., age, receptor status, metastasis information),
2. Availability of the selected molecular/genomic profiles (as described below), and
3. Removal of duplicate or incomplete records. Cases are excluded if key outcome labels or required features are missing, or if records are inconsistent across files.

The TCGA data types used in this research include: (a) Clinical data, such as age, ER/PR/HER2 receptor status, and metastasis indicators; and (b) Genomic features, including gene expression profiles and copy number alteration (CNA) data, as available for the selected cases. No TCGA radiology images or whole-slide histopathology images are used in model training or testing. TCGA is utilized as a source of structured clinical and genomic features. All imaging modalities incorporated in the proposed framework (mammography, MRI, DBT, and thermography) are obtained from the private dataset.

The ground-truth labels for TCGA samples are derived from clinical annotations, pathology reports, and staging fields, and are mapped to the study tasks described in “TCGA cohort selection and data modalities” section. The final TCGA split followed the same data partitioning strategy outlined in “Datasets integration and preprocessing” section (70% training, 15% validation, and 15% testing), and the evaluation protocol described in “Experimental design” section (Experimental Design) (Table 2).

**Table 2:** TCGA data used in this study (after filtering).

Item	TCGA project	Included cases	Excluded cases	Clinical variables	Genomic variables	TCGA imaging
Description	TCGA-BRCA	N_TCGA_	N_EXCL_	Age at diagnosis; ER status; PR status; HER2 status; TNM stage; lymph node involvement; metastasis status; treatment history	Gene expression (RNA-seq); Copy number alteration (CNA)	None

### Datasets integration and preprocessing

In the integration of datasets, the first TCGA^32^ dataset is used. The TCGA dataset has been widely used in the classification of breast cancer. The researchers take advantage of genomic and clinical data from TCGA to gain insight into molecular subtypes of breast cancer, identify genetic modifications associated with different subtypes, and develop classification models for improved diagnosis. From the TCGA dataset, genomic and clinical features can be extracted. Genomic features include somatic mutations, gene expression profiles, and clinical features such as age, gender, treatment history, oestrogen and progesterone receptor (EPR), and metastasis. The summary of the TCGA dataset is given in Table 3.

**Table 3:** Summary of the TCGA dataset.

Type	Subtype	Cancer types	Description	Format/notes
Clinical	Clinical data	All	Available clinical information	XML (per patient), tab-delimited TXT (grouped “biotab” per cancer type)
Biospecimen data	All	Information on sample processing	XML, tab-delimited TXT	grouped “biotab” per cancer type)
	Diagnostic image	All	Whole slide images of tissue for diagnosis	SVS
Imaging	Tissue image	All	Whole slide images of tissue samples for analysis	SVS
	Radiological image	Some	Pre-surgical radiological imaging (select cases)	DCM
DNA	All	Whole genome sequencing (select cases)	BAM, VCF, MAF (mutation calls)	Germline mutations controlled-access

To address inter-dataset variability between publicly available dataset and the private clinical dataset, dataset-specific preprocessing and normalisation strategies are applied. Imaging modalities are normalized independently using consistent resizing and standardization procedures. Moreover, structured clinical and genomic features from TCGA are processed through a dedicated multilayer perceptron (MLP) branch, separate from the imaging sub-networks. Since no patient overlap exists between datasets, integration is performed at the feature level rather than through direct record matching.

#### Description of the private clinical dataset

**Fig. 3:**
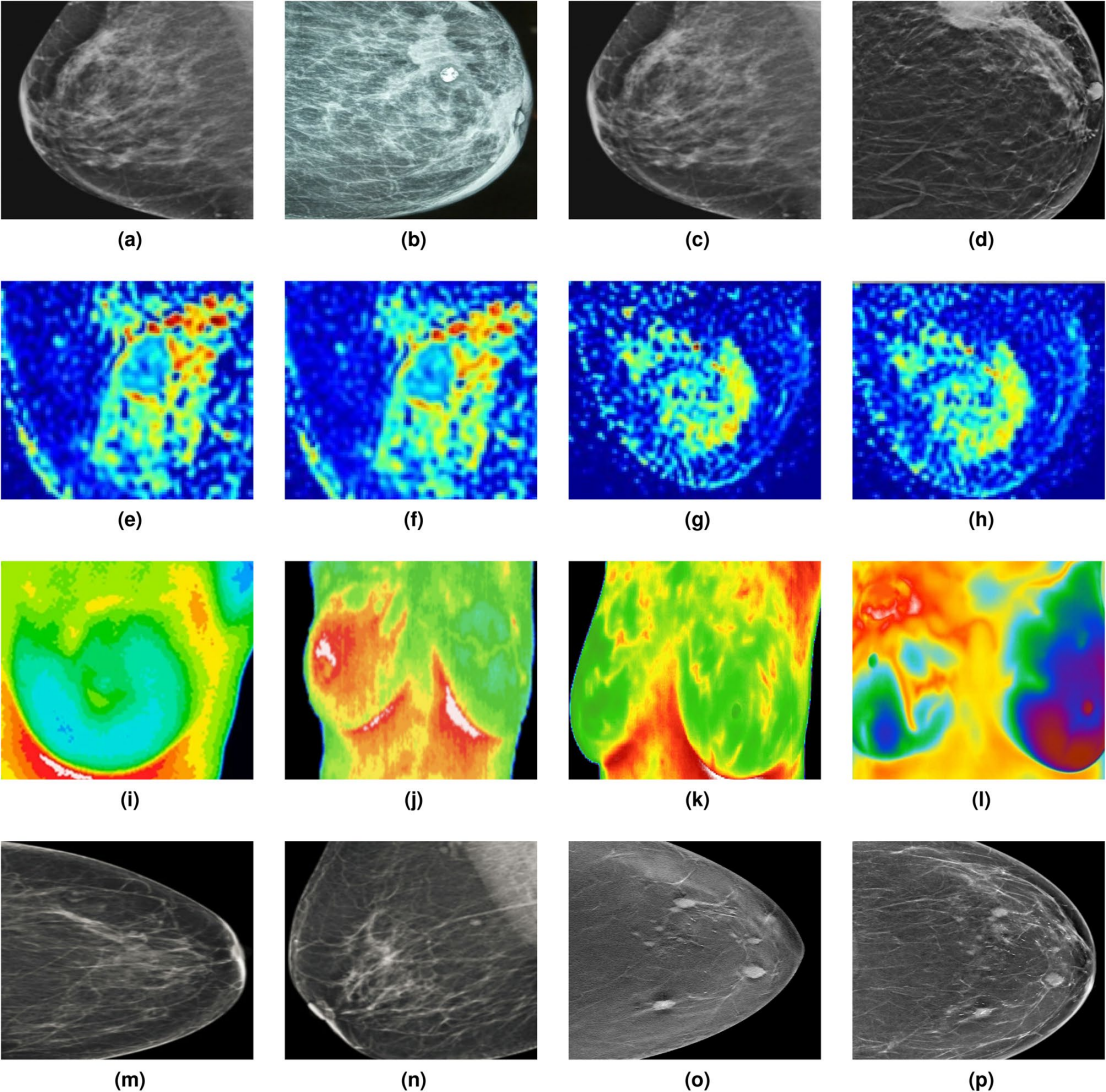
The private dataset contains different types of images: Row 1 (**a**–**d**) Grayscale mammography, Row 2 (**e**–**h**) $\rightarrow$ MRI (contrast-enhanced heatmap-like images), Row 3 (**i**–**l**) $\rightarrow$ Thermography (thermal color maps), and Row 4 (**m**–**p**) $\rightarrow$ Digital Breast Tomosynthesis (DBT).

The other dataset used in the proposed research is the private dataset. The private dataset contains different types of images, including mammography, MRI, digital breast tomosynthesis (DBT) and thermography, as shown in Fig. 3. The detail of the private dataset is given in Table 4. The ’Normal patients’ category in Table 4 consists of screening-negative individuals who showed no radiological evidence of breast malignancy at the time of imaging. These cases are confirmed as non-cancer through radiology reports and follow-up clinical evaluation. None of the cases are histopathologically confirmed as malignant. Images for this group are acquired using the same imaging protocols (mammography, MRI, DBT, or thermography) as applied in routine clinical practice. All normal cases are reviewed by qualified radiologists to ensure the absence of suspicious lesions.

**Table 4:** Distribution of samples used for hierarchical classification (normal vs cancer; Stage II vs Stage III).

Stages	Type	Age group	No. of samples	%age of samples
Stage II	Benign	18-20	167	3.27
		21-25	193	3.78
		26-30	266	5.22
		31-35	373	7.32
		36-40+	393	7.71
	Malignant	18-20	179	3.86
		21-25	166	3.25
		26-30	293	5.75
		31-35	261	5.12
		36-40+	461	9.04
	Total		1392	27.32
Stage III	Benign	18-20	173	2.68
		21-25	201	3.94
		26-30	209	4.10
		31-35	319	6.26
		36-40+	299	5.86
	Malignant	18-20	236	4.63
		21-25	189	3.71
		26-30	347	7.34
		31-35	199	3.90
		36-40+	397	7.79
	Total		1360	26.69
Normal patients		18-20	378	7.42
		21-25	296	5.81
		26-30	593	11.64
		31-35	378	7.42
		36-40+	597	11.71
	Total		2342	45.97
	Sub-total		5094	

To ensure reproducibility, a few technical details are applied during data processing and model training. Genomic data is first transformed using $log_2$ and then Z-score normalized^33^. For clinical data, missing numbers are filled in using the median, and missing categories are filled using the most common value (mode). The model is trained using the Adam optimizer, with a learning rate of 0.0001, a batch size of 32, and run for up to 50 epochs. Moreover, early stopping is also used to avoid overfitting. To make sure results are consistent every time, a random seeds is set throughout the process.

#### Private dataset description and labeling protocol

The private dataset is collected from Green Valley General Hospital, Pakistan, between the years 2023 and 2025. All data collection procedures are approved by the review board, and informed consent is obtained from all patients. All patient data is anonymized to ensure confidentiality and compliance with ethical standards, as detailed in the Ethics Statement.

- **Imaging modalities: ** The private dataset includes mammography, MRI, digital breast tomosynthesis (DBT), and thermography images. Not all patients underwent all imaging modalities; the available modalities varied depending on clinical indication and diagnostic workflow. Images are treated as independent inputs during model training, while labels are assigned at the patient level.
- **Ground-truth labeling: ** Diagnostic labels (benign or malignant) are established based on histopathological examination following biopsy or surgical resection. Cancer staging (Stage II or Stage III) is determined according to the TNM classification system documented in pathology and clinical reports.
- **Label granularity: ** Labels are assigned at the patient level and inherited by all corresponding images for that patient. On average, each patient contributed approximately 4 images, depending on modality availability.
- **Clinical validation: ** Diagnoses and staging were confirmed by at least two experienced oncologists and radiologists. In cases of disagreement, a consensus review is conducted. Inter-observer agreement is assessed using Cohen’s kappa coefficient and demonstrated substantial agreement ($\kappa = 0.82$).

#### Relationship between TCGA and the private multi-modal imaging dataset

TCGA and the private dataset are used as complementary data sources and are not linked at the patient level. Due to the absence of shared patient identifiers across datasets, alignment is performed at the feature/model level rather than direct case matching. Specifically, TCGA contributed structured clinical/genomic representations, while the private dataset contributed multi-modal imaging (mammography, MRI, DBT, and thermography). The proposed framework learns robust representations across these heterogeneous inputs through transfer learning and fusion, and performance is reported according to the clearly defined tasks in “TCGA cohort selection and data modalities” section.

Moreover, the impact of utilizing multiple heterogeneous datasets is improved model generalisation through exposure to diverse imaging characteristics, patient demographics, and feature distributions. Unlike single-dataset approaches that may learn dataset-specific patterns, the proposed multimodal integration approach reduces overfitting and enhances robustness to distributional variations across institutions.

### Features used in the proposed model

**Fig. 4:**
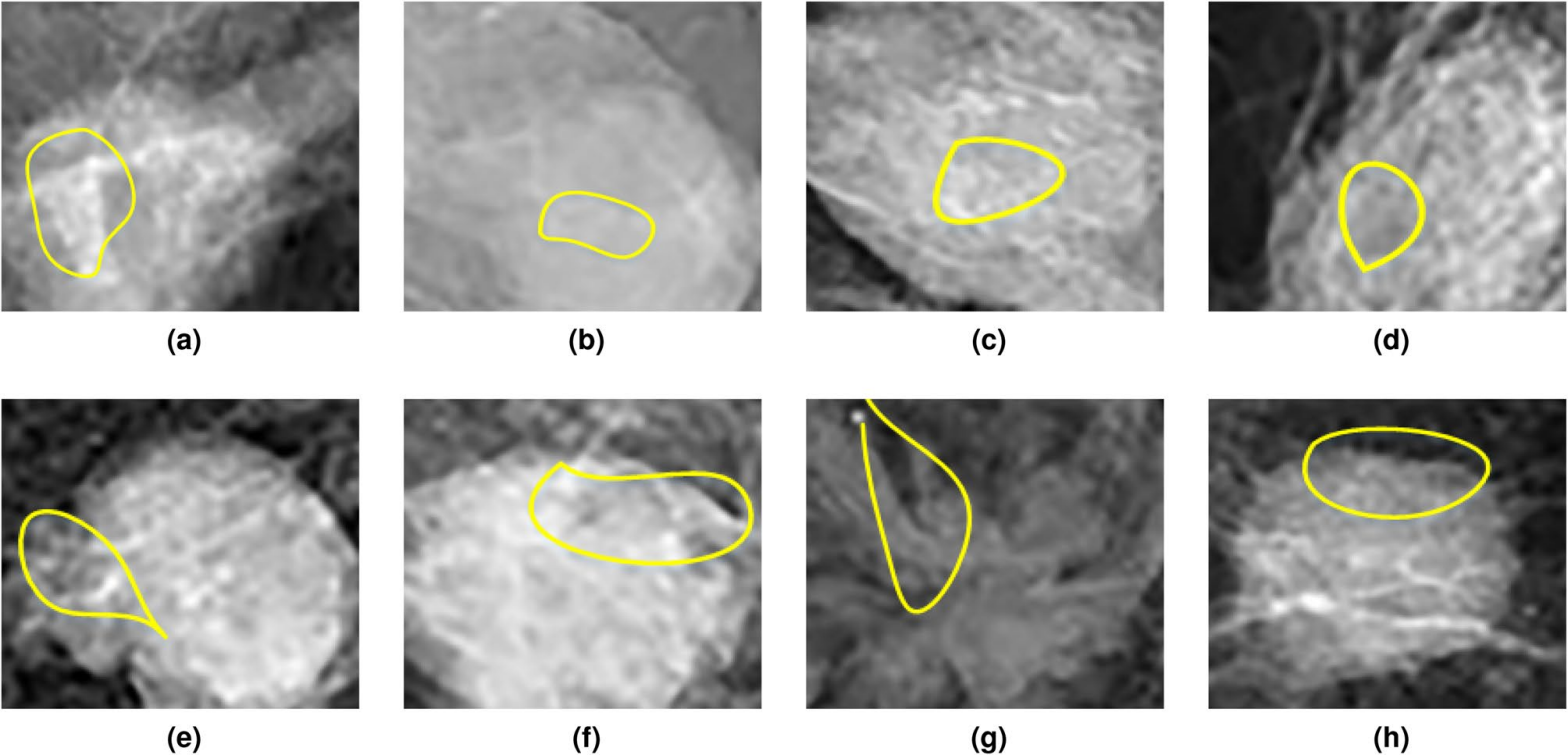
Representative mammography images with annotated lesion regions (highlighted in yellow). The first row (**a**–**d**) shows examples of benign cases, the second row (**e**–**h**) illustrates malignant tumor regions.

Table 5 shows the different features that are used in the proposed work for the detection of early detection types, i.e., benign or malignant breast tumors. Moreover, the state of each feature is provided in Table 5, which describes whether the woman is suffering from a breast tumour or a malignant breast tumour. Figure 4 shows the different masses that can appear in benign and malignant cases.

**Table 5:** Features used in the proposed work for classification of Stage II and Stage III breast cancer.

Feature	Stage II	Stage III
Tumor size	2–5 cm without lymph node involvement	>5 cm or tumor of any size with extensive lymph node involvement
Lymph node involvement	1–3 axillary lymph nodes affected	4 or more lymph nodes affected or fixed/matted nodes
Histological grade	Moderate (Grade 2)	High (Grade 3)
Ki-67 proliferation index	Moderate (10–20%)	High (>20%)
Estrogen receptor (ER) status	Often positive	Can be positive or negative
Progesterone receptor (PR) status	Often positive	May be reduced or negative
HER2 status	Variable	May be amplified (positive)
Distant metastasis	Not present	Rare, but higher risk than Stage II
Skin or chest wall involvement	Absent	May be present (e.g., skin ulceration, edema)
Genetic markers (e.g., BRCA1/2)	May or may not be present	Higher likelihood in aggressive cases
Imaging characteristics	Clear tumor boundary on imaging	Irregular margins, possible invasion to adjacent tissue
Treatment strategy	Surgery + radiation or chemotherapy	Multi-modal: surgery, radiation, chemo, targeted therapy

### Deep/machine learning algorithms used in the proposed work

Several algorithms have been explored in the literature for the detection of breast cancer by different researchers^17,34,35^. In this proposed work, the focus for early breast cancer detection lies in the use of a deep learning algorithm called the Deep Neural Network (DNN). Additionally, various machine learning algorithms, including support vector machine (SVM), decision tree (DT), and Naive Bayes(NB), are employed to compare the performance of both deep learning and machine learning approaches.

#### Fully connected deep neural network for structured TCGA data

This subsection describes the fully connected deep neural network (MLP) used for structured TCGA clinical and genomic features, which differs from the convolutional neural networks employed for imaging modalities.

In this research, the detection of human breast cancer is forecasted using a DNN. DNN architectures establish a hierarchical structure through hidden layers, where higher-level features are implicitly derived by combining lower-level features from each layer. The DNN model comprises an input layer, different numbers of hidden layers such aa s convolutional layer, a pooling layer, a dense layer, and a last output layer. The input layer is represented by an input vector *y*. The output $L^{k}_i$ for layer *k*, including units *i*, is computed as the weighted sum of the outputs of the preceding layer $L^{k-1}$ (particularly $B^0 = y$).

1 $$\begin{aligned} h^k= & X^kL^{k-1} + c^k, \hspace{20pt} 1\le k\le N \end{aligned}$$

2 $$\begin{aligned} L^k= & f(h^k) \end{aligned}$$

Where, $X^k$ represents the weight matrix corresponding to the relation between the $(k-1)^{th}$ layer and the $k^{th}$ layer. The bias vector for the $k^{th}$ layer is denoted as $c^k$. The total number of layers is denoted by *N* (in this case, $N = 5$, including the output layer). The activation function $f(\cdot )$ is the hyperbolic tangent (*tanh*), which is applied to the hidden units, allowing the neural network to naturally capture nonlinear relationships within the data. Additionally, the softmax function^36^ is employed as the activation function for the output layer ($N^{th}$ layer) in the DNN structure and is defined in Eq. (3):

3 $$\begin{aligned} \Xi ^{N} = \frac{exp(L^N)}{\sum _{i}exp\big ( h^{N}_i\big )} \end{aligned}$$

Subsequently, the weight initialization is commence between each layer employing the normalised initialisation method proposed by Glorot and Bengio^37^. The biases are set with moderate initial values, such as 0.1. The weights linking the layers are initialised from a truncated distribution, as specified in Eq. (4):

4 $$\begin{aligned} X \approx U \Bigg [ - \sqrt{\frac{2}{n_j + n_p}, \frac{2}{n_j + n_p}} \Bigg ] \end{aligned}$$

Here, $n_j$ and $n_p$ represent the number of input and output units, respectively. The proposed study on the binary classification task to predict breast cancer prognosis (long-term survival and short-term survival). To serve as the objective function for the final output layer of the Deep Neural Network (DNN) model, a cross-entropy loss is adopted. To mitigate the risk of overfitting, L2 regularisation is incorporated into the loss function, a technique widely employed in deep learning study^38^. Ultimately, the proposed DNN method is designed to minimize the loss function, and it is expressed in Eq. (5).

5 $$\begin{aligned} \mathcal {L}(z, \hat{z}) = -\frac{1}{N} \sum _{j=1}^{N} \left[ z_j \log (\hat{z}_j) + (1 - z_j)\log (1 - \hat{z}_j) \right] + \lambda \sum _{k=1}^{K} \Vert W_k \Vert _2^2 \end{aligned}$$

Here, $\lambda$ denotes the L2 regularisation coefficient, and $\Vert W_k\Vert _2^2$ represents the squared L2 norm of the weight matrix corresponding to the $k^{\text {th}}$ layer. The inclusion of the L2 regularisation term penalizes large weight magnitudes, thereby mitigating overfitting and enhancing the generalisation performance of the proposed model. Moreover, $z_j$ represents the true label of the $j^{\text {th}}$ sample, and $\hat{z}_j$ denotes the predicted probability obtained from the output layer. *N* denotes the batch size. $W_k$ represents the weight matrix of the $k^{\text {th}}$ layer, and *K* denotes the total number of weight matrices in the DNN model. In the proposed architecture, $K = 5$.

A prevalent challenge in training a Deep Neural Network (DNN) model is referred to as “internal-covariate-shift,” wherein the input distributions undergo changes in each layer during training due to parameter updates from preceding layers. In 2015, Google introduced a pioneering technique called batch normalisation^39^ to address this issue. Batch normalization enables the use of higher learning rates and reduces the need for meticulous weight initialisation. As anticipated, the incorporation of batch normalisation proves highly consequential in optimising our DNN model and achieving favorable results. To outline, the DNN model employed in the proposed search comprises one input layer, three hidden layers, and an output layer. Batch normalisation is applied to all five layers. To identify the optimal parameters, the proposed research adopts a grid search strategy outlined by Chen et al.^40^. Specifically, we explore the number of hidden layers, ranging from 1 to 4 in increments of 1, with each hidden layer containing 150, 450, 960, or 2500 units. The search for the optimal mini-batch size spans values from 32 to 128, with a step size of 32. The initial learning rate is chosen from 0.1 to 0.00010 with a magnification of $\times 0.1$. The selection of optimal parameters is based on the combination of parameters that yields the best performance, as measured by the value of the area under the curve (AUC)^41^. Ultimately, the best performance is achieved with a parameter combination featuring three hidden layers with 950, 460, 460, and 90 units, while the mini-batch size and initial learning rate are set to 32 and 0.010, respectively. The detailed parameter lists employed in our DNN model are provided in Table 6.

**Table 6:** Parameter lists employed in our DNN model.

No, of hidden layers	03
Hidden units in hidden layers	$1^{st}$ layer: 950, $2^{nd}$ layer: 460, $3^{rd}$ layer: 460, $4^{th}$ layer: 90
Starting learning rate	0.010
Mini batch size	32
Weight epoch	5-50
Batch normalisation epsilon	$\Bigg [ - \sqrt{\frac{2}{n_j + n_p}, \frac{2}{n_j + n_p}} \Bigg ]$
Activation function	Hyper tangent
Loss function error	Mentioned in Eq. (5)

#### Multi DNN model with multiple datasets (MDNNMDs)

**Fig. 5:**
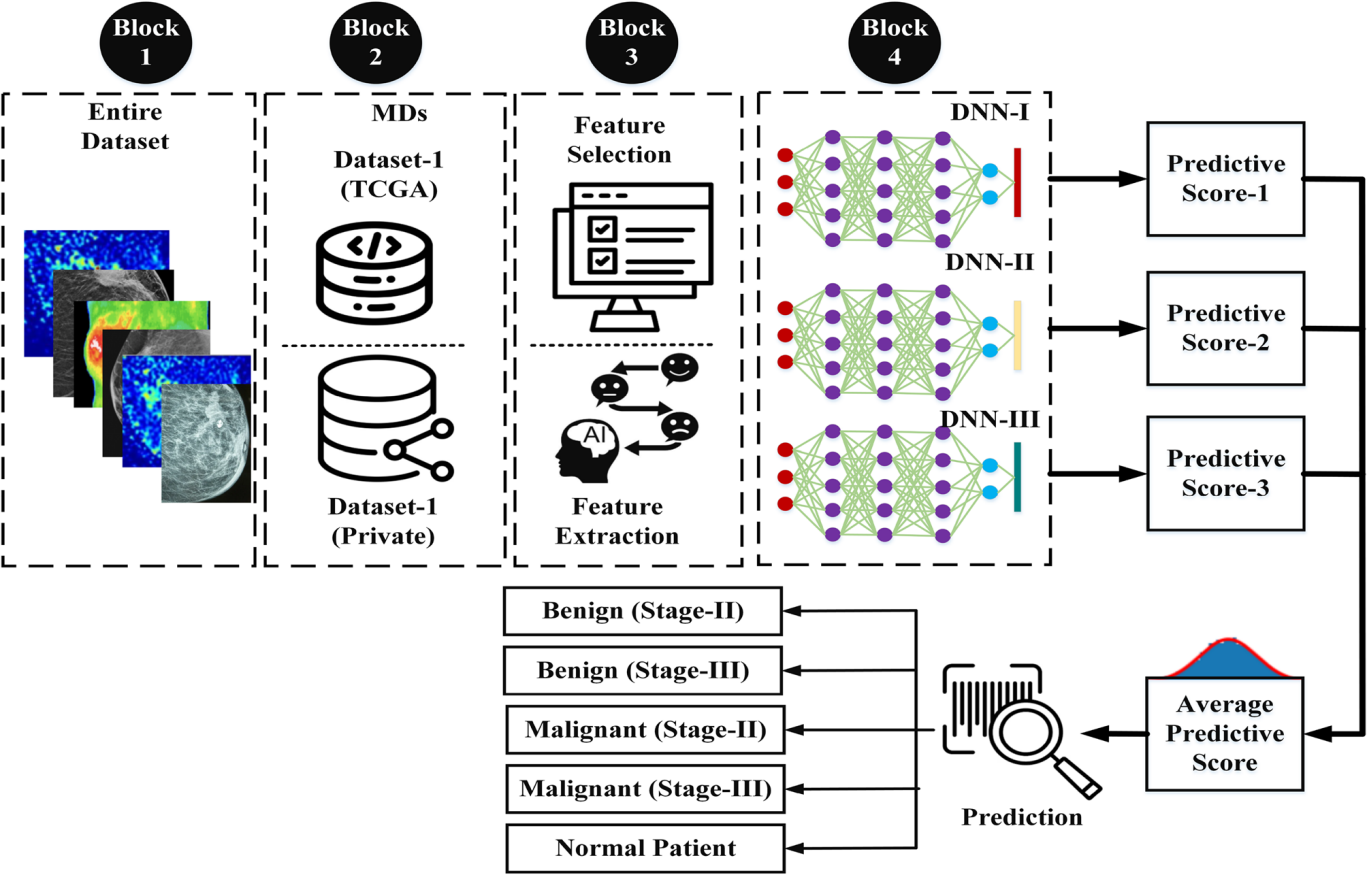
The generic structure of the proposed MDNNMDs.

A critical challenge in the proposed investigation involves the integration of multiple datasets (MDs) that incorporate mammography, MRI, digital breast tomosynthesis (DBT), and thermograph images. While a simple approach for discriminative tasks is to train a single deep neural network (DNN) model for MDs, this may prove inefficient due to variations in feature representation across different data sources^42^. To tackle this issue, a multi-DNN model is proposed that adeptly combines MDs. The structure of the MDNNMds method is shown in Fig. 5.

All images from the private dataset (mammography, MRI, DBT, and thermography) are resized to $512\times 514$ pixels prior to model input. Grayscale modalities are converted to a 3-channel format by channel replication to match the CNN input requirements. Initially, preprocessing techniques such as normalisation^43^, image filtering^44^, and image scaling^45^ are applied to MDs related to breast cancer data. In addition to imaging-based preprocessing, structured clinical and genomic attributes are also standardised and encoded prior to model integration.

Let $\textbf{x} \in \mathbb {R}^d$ denote the structured feature vector extracted from clinical and genomic attributes listed in Table 2, where *d* represents the number of selected features. Continuous variables were normalised using z-score standardisation, while categorical variables (e.g., receptor status) were encoded using one-hot encoding.

The normalised feature vector is passed through a multilayer perceptron (MLP) defined as:

6 $$\begin{aligned} \textbf{h}^{(1)}= & \sigma \left( \textbf{W}^{(1)} \textbf{x} + \textbf{b}^{(1)} \right) , \end{aligned}$$

7 $$\begin{aligned} \textbf{h}^{(l)}= & \sigma \left( \textbf{W}^{(l)} \textbf{h}^{(l-1)} + \textbf{b}^{(l)} \right) , \end{aligned}$$

where $\textbf{W}^{(l)}$ and $\textbf{b}^{(l)}$ denote the weight matrix and bias vector of layer *l*, and $\sigma (\cdot )$ represents the nonlinear activation function (tanh/ReLU).

The final structured branch output is computed as:

8 $$\begin{aligned} \hat{\textbf{y}}_{\text {struct}} = \text {softmax} \left( \textbf{W}^{(L)} \textbf{h}^{(L-1)} + \textbf{b}^{(L)} \right) , \end{aligned}$$

which produces class probability estimates that are later integrated with imaging-based predictions using the fusion strategy defined in Eq. (9). The fusion coefficients are empirically determined through validation-based grid search within the constraint $\alpha + \beta + \gamma = 1$. The threshold value (e.g., 0.25) was selected to ensure balanced contribution among modalities while avoiding dominance of any single branch. Sensitivity analysis on the validation subset confirmed that small coefficient variations did not significantly affect performance stability.

9 $$\begin{aligned} \begin{aligned} O_{\text {MDNNMDs}}&= \beta \cdot O_{\text {DNN-Expr}} + \gamma \cdot O_{\text {DNN-CNA}} + \alpha \cdot O_{\text {DNN-breast\_cancer}} \\ \text {subject to: }&\beta + \gamma + \alpha = 1, \quad \beta \ge 0,\; \gamma \ge 0,\; \alpha \ge 0 \end{aligned} \end{aligned}$$

Here, the parameters $\beta$, $\gamma$, and $\alpha$ represent three weight coefficients used to balance the contribution of each DNN model. The structured MLP branch consists of three hidden layers with 128, 64, and 32 neurons, respectively. Each hidden layer is followed by a nonlinear activation function (tanh/ReLU) and dropout regularisation. Batch normalization is applied after each dense layer to improve training stability.

A summary of the overall MDNNMDs architecture, including the imaging sub-networks, structured TCGA branch, and fusion strategy, is provided in Table 7.

**Table 7:** Unified architecture summary of the proposed MDNNMDs framework.

Component	Architecture description
Imaging sub-network (per modality)	ResNet-50 (pretrained) + GAP + FC + Dropout + Softmax
Input size (images)	$224 \times 224 \times 3$
TCGA structured branch	MLP with fully connected layers + ReLU + Dropout
Activation function (MLP)	ReLU / Tanh
Regularization	L2 regularization + Dropout
Fusion method	Score-level weighted aggregation (Eq. 6)
Output layer	Softmax classifier

Subsequently, a feature reduction method such as principle component analysis^46^ is employed to reduce the number of variables in breast cancer data. The number of principal components retained is selected to preserve 95% of the cumulative variance, ensuring minimal information loss while reducing computational complexity. PCA is chosen due to its effectiveness in handling correlated features and its computational efficiency compared to nonlinear alternatives such as t-SNE or autoencoder-based reduction, which are typically more suitable for visualisation rather than classification pipelines.

In the third step, a quadruple-modal DNN is introduced to effectively extract information from the big data. Consequently, individual DNN models are trained, each corresponding to a specific sub-data category, in a greedy manner. Finally, the proposed model performs a score-level fusion from each independent model. The final output of MDNNMDs, determined through a weighted linear aggregation^47^, is computed using Eq. (9). Moreover, fusion is performed at the score level (i.e., combining predicted probability scores from each sub-network) rather than concatenating intermediate feature vectors.

In our implementation, each imaging modality is processed using a ResNet-50 backbone pretrained on ImageNet, followed by global average pooling, a fully connected layer with dropout, and a final softmax layer for classification. Transfer learning and fine-tuning details are provided in “Transfer learning” and “Fine tuning” sections. In parallel, TCGA clinical and genomic features (clinical variables, gene expression, and CNA data) are processed through a multilayer perceptron (MLP) consisting of fully connected layers with non-linear activation and dropout. This produces corresponding class probabilities. The outputs from all sub-networks are combined using a score-level fusion strategy, where the final prediction is obtained through weighted linear aggregation of probability scores as defined in Eq. (9), with fusion weights $(\alpha ,\beta ,\gamma )$ optimized on the validation subset within each fold (Table 8).

**Table 8:** Summary of MDNNMDs implementation details for reproducibility.

Component	Specification
Image input size	$224 \times 224 \times 3$
Modalities (private data)	Mammography, MRI, DBT, Thermography
Normalisation	[0, 1] scaling + z-score (train-split statistics only)
Backbone (per modality)	ResNet-50 (ImageNet pretrained)
Head (per modality)	GAP + Dense + Dropout + Softmax
TCGA branch	MLP on clinical + gene expression + CNA features
Fusion	Score-level weighted fusion
Training	Adam, batch size 32, learning rate $1\times 10^{-4}$, early stopping

**Fig. 6:**
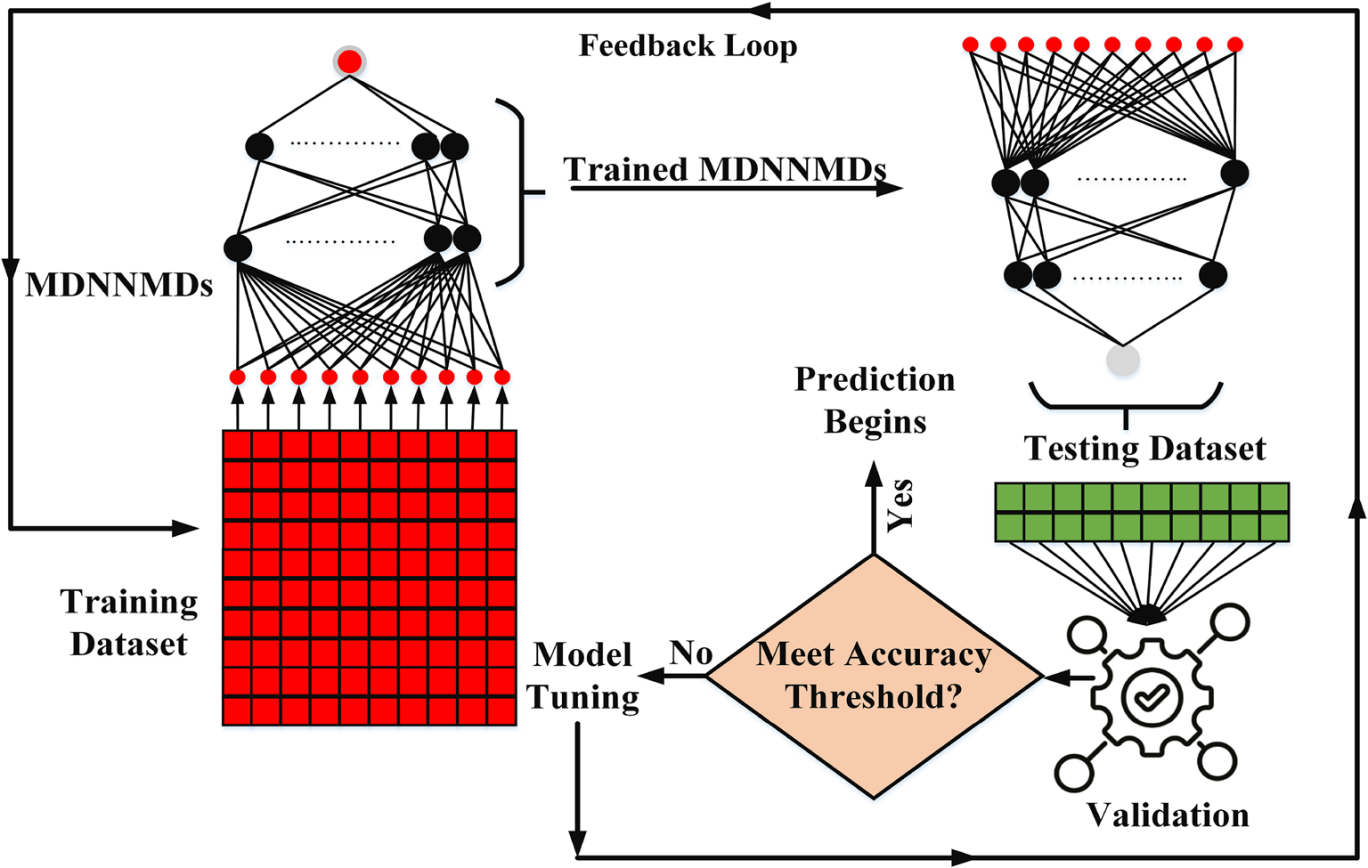
Training and testing process of the MDNNMDs.

In the proposed research, MDNNMDs optimizes the parameters $\beta$ $\gamma$ and $\alpha$ for the sub-DNN models based on the best prediction performance determined using the validation set (see Experimental Design). Various combinations of $\beta$ $\gamma$, and $\alpha$ are screened with a step of 0.25, and ultimately, the values $\beta$ = 0.19, $\gamma$ = 0.8, and $\alpha$ = 0.27 are selected for the Mds dataset. The implementation of MDNNMDs is based on the Keras and TensorFlow deep learning library, open-source software library for Machine Intelligence. Training is carried out using two Nvidia GTX TITAN Z graphics cards. The training and testing process of MDNNMDs is shown in Fig. 6.

#### Transfer learning

Transfer learning is a form of machine learning in which a model is trained to perform prediction tasks, such as detecting breast cancer, COVID^48^, and pneumonia^49^. The ResNet-50 backbone is initialized with ImageNet-pretrained weights. Only the final classification head is randomly initialized, while convolutional layers retain pretrained parameters during initial transfer learning. Gradual unfreezing is applied during fine-tuning. This approach is useful when the dataset used to retrain the existing model is smaller than the original dataset. The proposed research introduces a method for repurposing an ResNet model, initially trained on the ImageNet dataset, to acquire training features on a different dataset, namely BreakHis and INbreast. By employing transfer learning, capitalizing the features learned from the MDs dataset and adapting the features and the structure of the model to the new dataset to avoid the need to initiate the learning process from scratch with random weight initialisation.

#### Fine tuning

The fine-tuning of a network is based on the principles of transfer learning^50^. Initially, a Deep Neural Network (DNN) is trained to acquire features for a broad domain by optimizing the classification function to minimize errors within that domain. Subsequently, the classification function is substituted, and the network undergoes further adjustments to minimize errors within a more specific domain. This configuration facilitates the migration of the network’s features and characteristics from a broad domain to a specialized domain.

The DNN employs the softmax classification function to determine the probability of each class in the MDs dataset. To initiate the fine-tuning process, the softmax classifier values are erased and replaced with new random values. The updated softmax classifier is then trained from the beginning, that utlizes the back-propagation methodology.

Accurate calibration of learning rates for each layer is essential before commencing the backpropagation process for fine-tuning. In the proposed research, the learning rate for the top classification layer is increased to 12 and reduced to 0.15 for the next three layers. The back-propagation approach was iterated two thousand times, employing stochastic gradient descent (SGD)^51^ to optimize the network parameters.

Model training is conducted in two stages. During initial training, the Adam optimizer is used with an initial learning rate of $1 \times 10^{-4}$ and a batch size of 32. Early stopping is applied based on validation loss with 50 epochs to prevent overfitting. The maximum number of training epochs is set to 200. During the fine-tuning phase, stochastic gradient descent (SGD) with momentum is employed with a reduced learning rate to refine pretrained weights. Learning rate adjustments are performed manually based on validation performance. Moreover, data augmentation is applied to imaging modalities during training, including random rotations, horizontal flips, and slight zoom transformations. Augmentation was restricted to the training subset only. Finally, to address potential class imbalance, stratified sampling is used during cross-validation to preserve class distribution across folds. No synthetic oversampling techniques are applied. The basic details of the machine learning algorithms used in the proposed work are given below.

### Support Vector Machine (SVM)

The SVM, is a technique that requires a dataset in order to do the desired task. In addition to this, it selects a sufficient number of samples, which are then transformed as a support vector, and produces a linear discriminant function. The SVM classifier seeks an optimal separating hyperplane defined in Eq. (10):

10 $$\begin{aligned} \textbf{w}^\top \textbf{x} + b = 0, \end{aligned}$$

where, $\textbf{w}$ is the weight vector and *b* is the bias term. The optimisation objective is given in Eq. (11):

11 $$\begin{aligned} \min _{\textbf{w}, b} \frac{1}{2} \Vert \textbf{w}\Vert ^2 + C \sum _{i=1}^{N} \xi _i, \end{aligned}$$

A radial basis function (RBF) kernel is used with regularization parameter $C=1.0$ and kernel coefficient $\gamma =0.01$, selected via grid search.

The use of SVM provides a solution to the problem of linear constraints^52^. In SVM, different classes may be linearly partitioned to exhibit the highest hyperplane margin. Following the selection of an appropriate mapping, the newly obtained samples appear to be linearly separable or capable of being linearly fitted in the high-level plane. SVM works to locate the hyperplane that brings the two groups to the closest feasible relationship^53^.

### Naive bayes

The NB method is proposed based on the application of Bayes’ theorem^54^. By employing Bayes’ theorem and following specific procedures outlined in^55^, enhancements can be made to the NB classifier. Our study concludes that “T” represents an instance within a training set, each marked with group labels denoted as $G_1, G_2, G_3, \cdots , G_k$. Each specimen is an entity of *n* dimensions expressed as the formula $F = f_1, f_2, f_3, \cdots , f_n$, where *f* encompasses the *n* features corresponding to its *n* dimensions. The NB classifier estimates posterior probability using Bayes’ theorem:

12 $$\begin{aligned} P(y|\textbf{x}) = \frac{P(\textbf{x}|y) P(y)}{P(\textbf{x})}. \end{aligned}$$

where $P(y|\textbf{x})$ denotes the posterior probability of class *y* given feature vector $\textbf{x}$, $P(\textbf{x}|y)$ represents the likelihood of observing features $\textbf{x}$ under class *y*, *P*(*y*) is the prior probability of class *y*, and $P(\textbf{x})$ is the marginal probability of the feature vector.

If the probability that group $G_i$ is associated with specimen *G* surpasses the probability of each of the other groups in relation to *G*, then the specimen *G* is deemed to belong to group *i*. This formal statement is expressed in Eq. (13).

13 $$\begin{aligned} {\left\{ \begin{array}{ll} P(G_i|F) > P(G_j|F) \hspace{20pt} & for 1\le j \ge k_j = i\\ P(G_i|F) = \frac{P(F|G_i)P(G_i)}{P(F)} & \text {Baye's theorem} \end{array}\right. } \end{aligned}$$

### Decision Tree

DT represents a supervised learning technique employed for the categorization of data into distinct groups. The size of the tree may exhibit a correlation with the number of features present in the dataset. In the growth of the tree, heuristics play a crucial role in computing costs and assessing classification performance^56^. Typically, decision trees utilize an impurity-based heuristic to calculate the purity of the resulting subset after applying the splitting feature to divide the training data^57^. To construct a tree for classification, the initial step involves selecting a root node, determined by computing the Information Gain (IG). The node with the highest IG is then chosen as the splitting attribute^58^. The computation of IG can be expressed as follows:

14 $$\begin{aligned} IG (A,B) = Entropy (A) - \sum _{b}\frac{|A_b|}{|A|}Entropy(A_b) \end{aligned}$$

Where *A* and *B* represent the feature vectors and features, respectively, *b* represents its value and $A_b$ represents the subset of *A* that contains occurrences of $B = b$. The entropy (A) may be calculated as follows:

15 $$\begin{aligned} Entropy (A) - \sum _{h=1}^{|X|} P_A (x_h)log P_A (x_h) \end{aligned}$$

Where $P_A (x_h)$ is the probability of events belonging to $x_h$ in *A* and |*X*| is the total number of categories in the dataset.

### Experimental design

All experiments are implemented using Python with the PyTorch deep learning framework. Training was performed on a workstation equipped with Windows 11, an Intel Core i7 processor, an NVIDIA RTX 5090 GPU (32 GB VRAM), and 64 GB of system memory. To ensure robust evaluation, a 5-fold cross-validation strategy is employed, in accordance with established practices in previous cancer prediction methodologies^59–61^. The dataset is randomly partitioned into five equal subsets. In each iteration, four subsets are used for model development, and one subset is reserved for testing. Within the development portion, the data is further split into 70% for training 15% for validation, and 15% for testing. This process is repeated across all five folds, and the final performance metrics are averaged over the folds.

Furthermore, in the proposed research, MDNNMDs do not simultaneously optimize model configurations and weight coefficients. Initially, various configurations are explored, and each sub-DNN is trained with a single-domain training set (weights and bias), mitigating overfitting through validation set usage. Subsequently, optimal configuration parameters are chosen based on the AUC value as the criterion. Finally, after training the sub-DNNs, different combinations of coefficients ($\alpha , \beta , \gamma$) are selected until the classification performance (AUC value) on the validation set reaches its maximum.

In assessing performance, a Receiver Operating Characteristic (ROC) curve is generated, depicting the relationship between sensitivity and 1-specificity across various decision thresholds, and the Area Under the Curve (AUC) is computed. Additionally, the evaluation metrics such as Accuracy ($A_{acc}$), Specificity ($S_{spe}$), Precision ($P_{pre}$), and Matthew’s Correlation Coefficient ($M_{Mcc}$) are utilized for performance evaluation and are defined by the equations given in Table 9.

**Table 9:** Parameters used in the evaluation of the proposed model.

Parameters	Mathematical equation	Parameters	Mathematical equations
Accuracy	$A_{Acc} = \frac{{T.P} + {T.F}}{T.P + T.F+ F.P + F.N}$	Precision	$P_{pre} = \frac{T.P}{T.P + F.P}$
Matthew’s	$M_{Mcc}$ =		
Correlation	$\frac{T.P \times T.N - F.P \times F.N}{\sqrt{(T.P + F.P)\times (T.P + F.N) \times (T.N + F.P) \times (T.N + F.N)}}$	Specificity	$S_{spe} = \frac{T.N}{T.N + F.P}$
Coefficient (MCC)			
Recall	$R_{Rcl} = \frac{T.P}{T.P + F.N}$	Fscore	$Fscore = 2 \times \Bigg [\frac{\times P_{pre} \times R_{Rcl}}{P_{pre} + R+{Rcl}}\Bigg ]$

Here, *T*.*P*, *T*.*N*, *F*.*P*,  and *F*.*N* represent true positives, true negatives, false positives, and false negatives, respectively.

#### Evaluation protocol and data splitting strategy

To ensure a fair comparison between the proposed FT-MDNNMDs framework and classical machine learning baselines (SVM, Decision Tree, and Naïve Bayes), identical preprocessing and data partitioning strategies are applied across all models. Imaging inputs underwent the same resizing, normalization, and feature extraction procedures before model training. For classical classifiers, deep features extracted from the final fully connected layer of the corresponding sub-network are used as input representations. Furthermore, all models are evaluated using the same patient-level stratified 5-fold cross-validation splits.

To prevent data leakage and ensure reliable performance estimation, all data splitting is performed at the patient level. Images belonging to the same patient are kept within the same fold and are never distributed across training, validation, and testing subsets. The TCGA dataset (clinical and genomic features) and the private imaging dataset are processed independently at the feature level and integrated during model training. As there is no patient overlap between TCGA and the private dataset, cross-dataset leakage is inherently avoided.

A stratified 5-fold cross-validation strategy is employed to preserve class distribution (Normal, Benign, Malignant, Stage II, and Stage III) across folds. The dataset is randomly partitioned into five mutually exclusive subsets at the patient level. In each iteration, four folds are used for model development, and one fold is reserved for testing. Within the development folds, the data are further divided into 70% training, 15% validation, and 15% testing subsets for hyperparameter tuning and early stopping. Patient identifiers are strictly enforced to ensure that no subject appears in both training and testing sets within any fold. For each fold, performance metrics including Accuracy, Precision, Recall, F1-score, Specificity, MCC, and AUC are computed on the test subset, and the final reported results correspond to the mean and standard deviation across all five folds.

To further ensure experimental integrity, we verified that no patient identifier appeared in both training and testing subsets across any fold. This limited patient-level separation eliminates the possibility of performance inflation due to data leakage. In addition to reporting mean performance values across folds, 95% confidence intervals (CIs) are computed for key metrics including Accuracy, AUC, Sensitivity, and Specificity, using standard deviation across folds.

### Machine learning algorithms for comparison

In order to validate the advantages of the proposed multimodal DNN, existing single-dimensional data-based methods for the prediction of early breast cancer are also investigated in this research. The key distinction between the existing methods and MDNNMD lies in the integration of *MDs*, as the existing methologied utilizes data of a single type.

To demonstrate the efficacy of MDNNMDs in predicting early breast cancer, four commonly used classifiers in human breast cancer prediction: SVM, RF, NB, and LR are used in comparison. Four baseline classifiers are selected as representative traditional machine learning approaches commonly used in medical image classification. More complex ensemble-based baselines are not included to maintain computational consistency and avoid overfitting risks on the relatively limited dataset size.

## Experimental results, analysis, and discussion

The experiments are conducted using Python 3.7 on a system equipped with an NVIDIA GeForce RTX GPU and 16GB of RAM. TensorFlow and PyTorch libraries were utilised, with Jupyter Notebook serving as the development environment.

Unless otherwise specified, the reported ROC curves, confusion matrices, and performance metrics correspond to Task 1 (Normal vs. Tumor classification). Stage-level and tumor-type classification results are indicated where presented.

The experimental workflow is conducted in three sequential stages. First, the ResNet-50 backbone is initialized with weights pretrained on the ImageNet dataset to provide general visual feature representations. Second, transfer learning is performed using publicly available breast imaging datasets (BreakHis and INbreast) to adapt the pretrained network to breast cancer–related visual patterns. Third, the model is fine-tuned and evaluated on the combined MDs dataset, consisting of TCGA structured clinical/genomic features and the private multi-modal radiology dataset (Tables 10, 11).

All performance metrics reported in Tables 12 and 13 correspond to the final evaluation stage on the MDs dataset under the 5-fold cross-validation protocol. BreakHis and INbreast are used only during the transfer-learning phase and are not included in the final performance evaluation. No domain adaptation techniques are applied when transferring between histopathology and radiology modalities; transfer learning is used only to improve feature initialization and convergence stability.

### Binary classification performance and ROC analysis

**Fig. 7:**
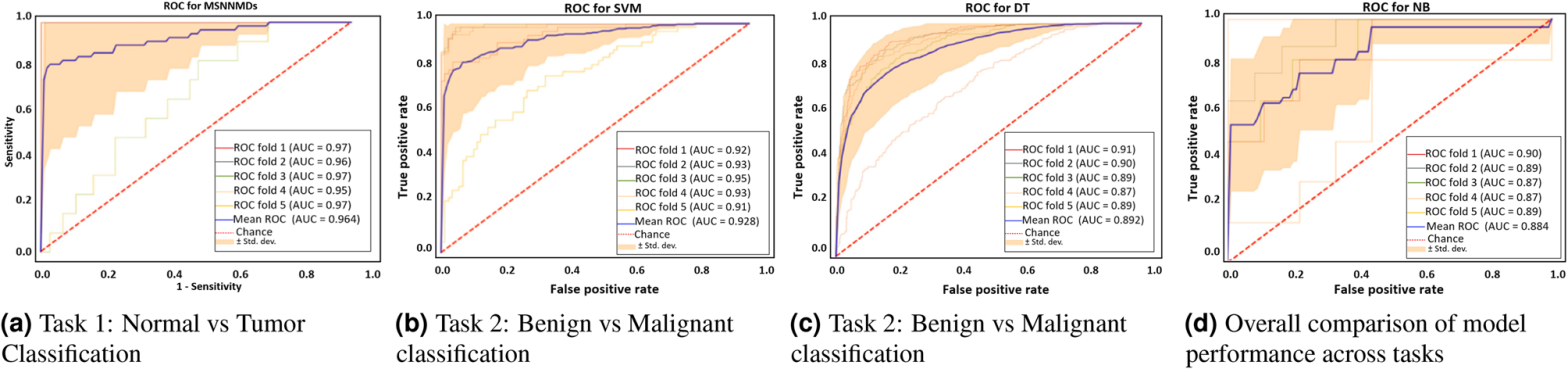
ROC curves for Task 1 (Normal vs Tumor classification) evaluated on the combined MDs dataset under 5-fold cross-validation. Sensitivity (True Positive Rate) is plotted against 1 – Specificity (False Positive Rate).

Figure 7 presents the ROC curves and corresponding AUC values under the 5-fold cross-validation protocol on the combined MDs dataset for the evaluated classification tasks. The curves plot Sensitivity (True Positive Rate) against 1 – Specificity (False Positive Rate), providing a comprehensive assessment of discriminative performance. As shown in Fig. 7a, the Normal vs Tumor classification task demonstrates the strongest performance, with a high mean AUC of 0.964 and fold-wise values ranging from 0.95 to 0.97. The narrow shaded regions indicate minimal variability across folds, confirming stable and reliable separation between cancerous and non-cancerous cases. Figure 7b illustrates the Benign vs Malignant classification task, which also achieves strong discriminative capability, although with slightly greater variability compared to Task 1. The AUC values remain consistently high, reflecting effective differentiation between tumor types.

In Fig. 7c, the Stage II vs Stage III classification task presents comparatively lower AUC values and slightly broader variability. This behavior is expected given the increased clinical similarity between adjacent cancer stages, which makes stage-level discrimination inherently more challenging. Finally, Fig. 7d provides an overall comparison of model performance across tasks, highlighting that the proposed framework maintains strong diagnostic capability across binary and stage-level classifications. The consistently high AUC values across tasks reinforce the robustness and generalization capability of the proposed FT-MDNNMDs model.

### Box plot analysis of model performance

**Fig. 8:**
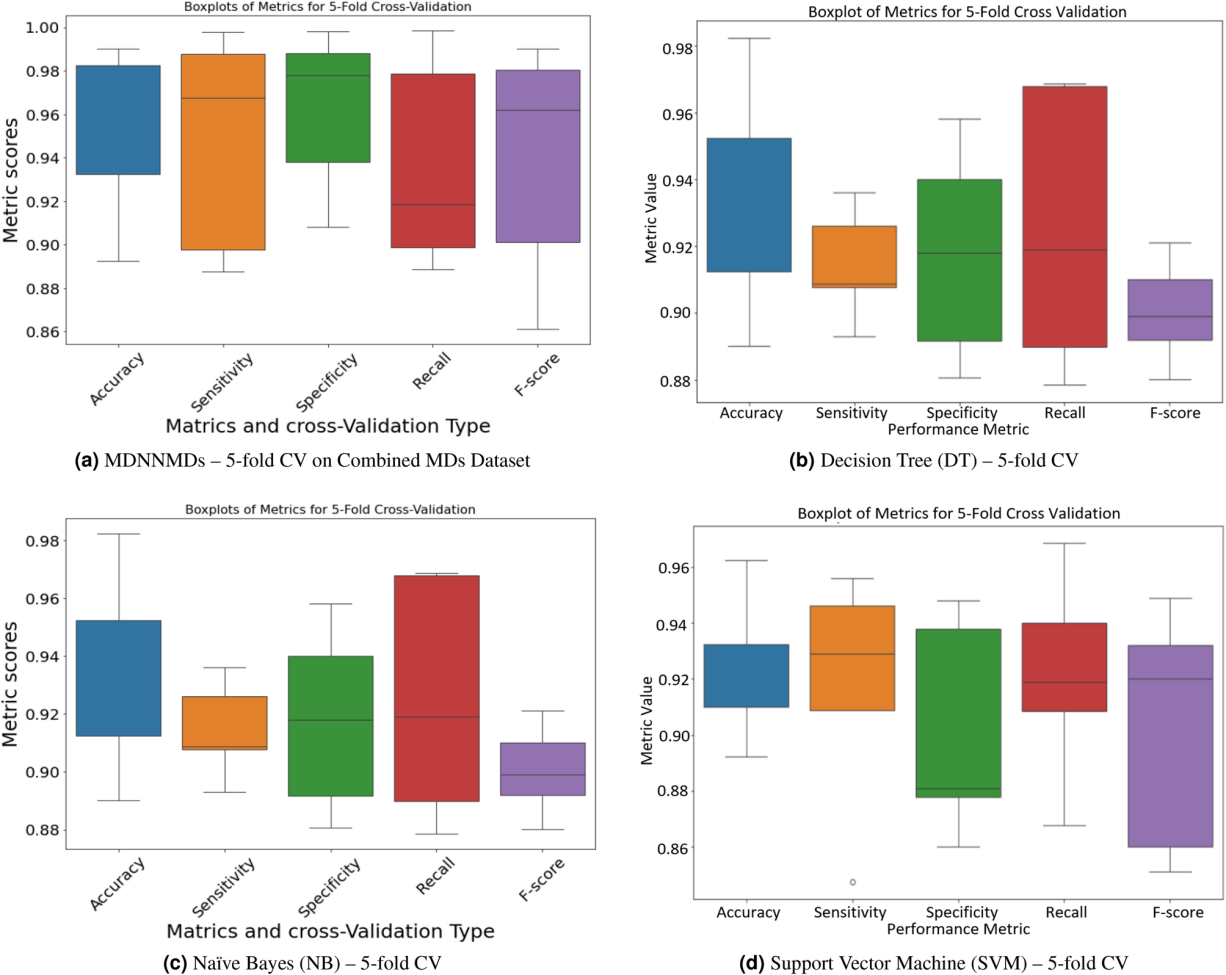
Box plots of performance metrics across 5-fold cross-validation on the combined MDs dataset.

Figure 8 presents box plots illustrating the distribution of key performance metrics–accuracy, sensitivity, specificity, recall, and F-score–across 5-fold cross-validation for the proposed MDNNMDs model and its variants integrated with SVM, DT, and NB classifiers. In Fig. 8a, the proposed MDNNMDs model exhibits tightly packed box plots with small interquartile ranges and consistently high median values across all five metrics. Accuracy and F-score maintain medians around 0.99, with all values above 0.98, reflecting highly stable classification performance. Sensitivity and recall show slightly broader ranges but still remain highly centered, indicating reliable detection of positive (malignant) cases. The limited presence of outliers further underscores the model’s robustness and consistency across folds. In contrast, Fig. 8b, showing results for the SVM-integrated variant, reveals greater variability. The sensitivity and F-score plots are particularly wide, with medians dropping below 0.94 and a noticeable spread from approximately 0.88 to 0.96. This indicates inconsistent identification of positive samples across folds and reduced generalizability. The accuracy and specificity, though relatively better, still exhibit more spread than MDNNMDs, implying that the SVM variant is more sensitive to training data variation.

Figure 8c, representing the DT-integrated variant, displays a broad distribution with several outliers, especially in the sensitivity and specificity metrics. The median sensitivity dips closer to 0.91, and the spread in accuracy and F-score ranges from around 0.88 to 0.95. These wide interquartile ranges suggest that decision trees, known for their tendency to overfit, may perform well in some folds but poorly in others, leading to overall performance instability. Lastly, Fig. 8d illustrates the NB-integrated variant, which demonstrates slightly better consistency than DT and SVM but still lags behind the proposed MDNNMDs. The box plots show moderate dispersion in sensitivity and F-score, with medians around 0.92 and wider whiskers indicating fluctuating performance. While accuracy and specificity remain reasonably high, the lower and more dispersed sensitivity values suggest a higher likelihood of false negatives. From such box plot analysis, it can be analyzed that the MDNNMDs model significantly outperforms its counterparts in terms of performance consistency, central tendency, and reliability across all key metrics which makes it a superior choice for early breast cancer classification.

### Impact of transfer learning and fine-tuning

**Fig. 9:**
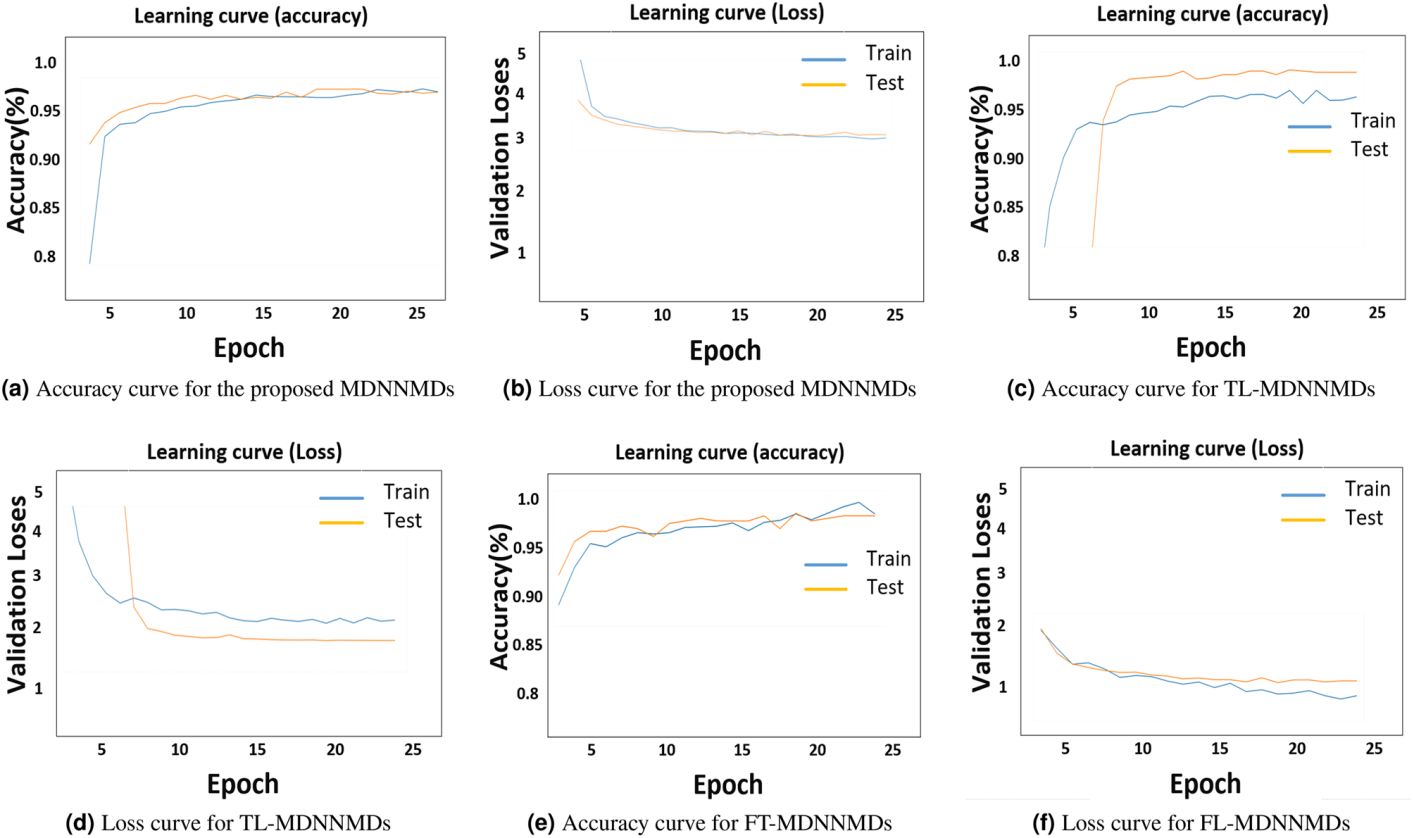
Learning curves in terms of accuracy and loss.

The model undergoes progressive enhancements through transfer learning and fine-tuning. Figure 9 provides a comprehensive view of the model’s performance progression through transfer learning (TL) and fine-tuning (FT), visualized via accuracy and loss learning curves over 25 training epochs.

In Fig. 9a and b, the proposed MDNNMDs model demonstrates strong learning behavior. The training and validation accuracy curves rapidly converge above 98%, with minimal gap, indicating excellent generalisation. The loss curves exhibit a consistent downward trend for both training and test sets, stabilizing below 1.5, reflecting a well-optimized model with minimal overfitting. However, Fig. 9c and d illustrate the learning curves after incorporating transfer learning (TL-MDNNMDs). Here, validation accuracy quickly escalates, surpassing 99% within the first few epochs. Although the training curve starts slightly lower than the test curve initially (a sign of beneficial transfer), both eventually stabilize around a high accuracy mark. The corresponding loss curves reinforce this trend, where test loss drops sharply and stabilizes around 1.4, demonstrating how TL aids rapid convergence and improved feature representation from pre-trained knowledge.

Further enhancement is evident in Fig. 9e and f, which show the results after fine-tuning (FT-MDNNMDs). The fine-tuned model achieves the highest validation accuracy, peaking at approximately 99.7%, with training accuracy closely aligned–signaling that the model has effectively captured task-specific patterns. Loss curves also show the most significant improvement here, with validation loss reducing further to 1.08%, highlighting better model calibration and reduced generalisation error. These improvements confirm the efficacy of integrating TL and FT into the MDNNMDs framework for superior performance in early breast cancer classification.

### Confusion matrix analysis

**Fig. 10:**
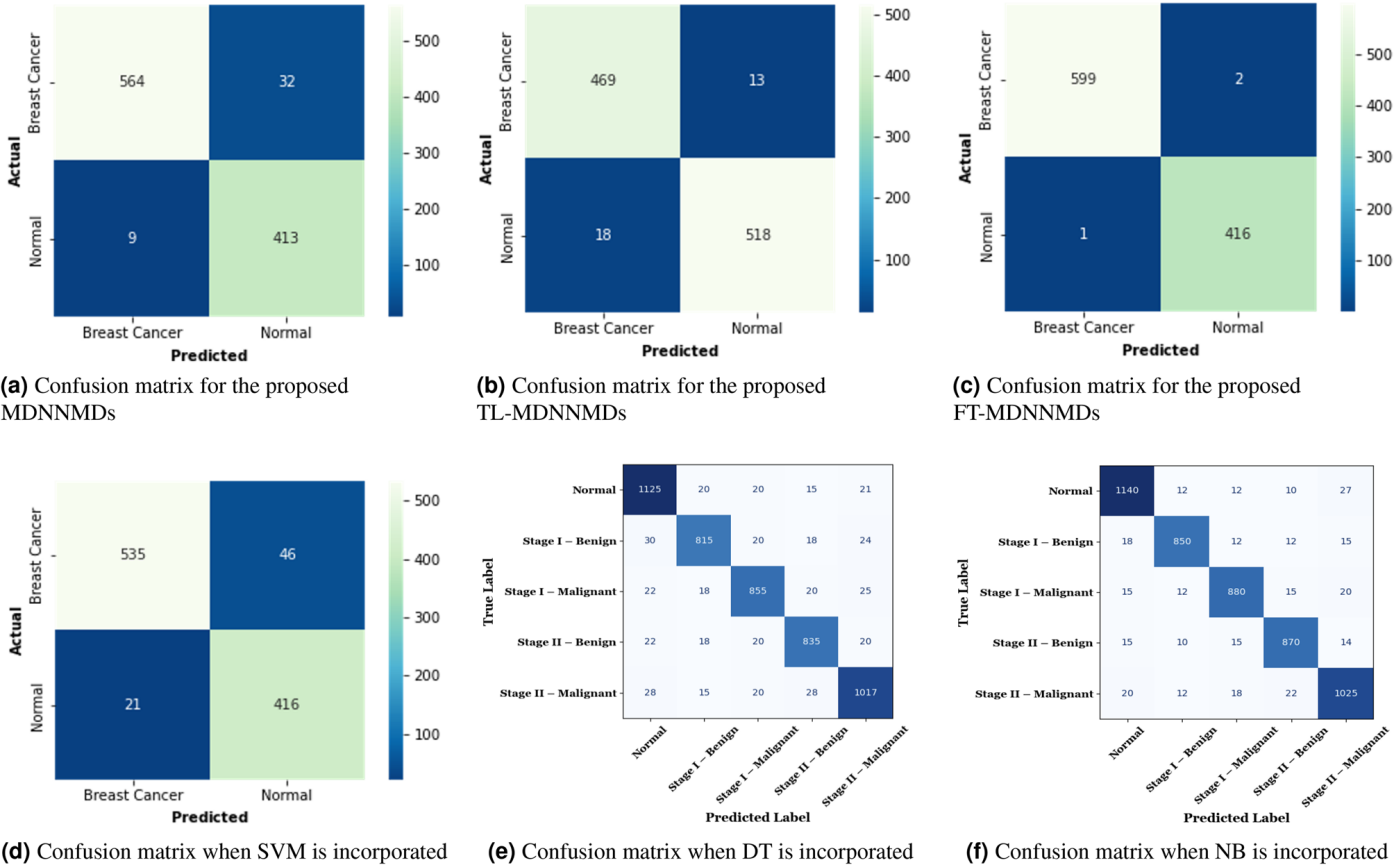
Confusion matrices.

The confusion matrices for the proposed MDNNMDs, TL-MDNNMDs, FT-MDNNMDs, and their counterparts incorporating traditional classifiers such as Support Vector Machines (SVM), Decision Trees (DT), and Naive Bayes (NB) are presented in Fig. 10. A detailed evaluation of these matrices reveals significant insights into each model’s classification capabilities for detecting breast cancer from medical imaging data.

Although the confusion matrices present in Fig. 10 show absolute classification counts, class-wise performance percentages are reflected in the reported sensitivity, specificity, precision, and recall metrics as shown in “Per-class performance analysis” section. These normalized measures provide proportional interpretation relative to true class distributions. This ensures an assessment of model performance without requiring additional normalized matrices.

### Comparative evaluation with existing methods

Table 12 presents a detailed statistical comparison of the proposed MDNNMDs, TL-MDNNMDs, and FT-MDNNMDs models against conventional classifiers such as SVM, Decision Tree (DT), and Naive Bayes (NB), as well as existing methodologies from prior studies. The proposed FT-MDNNMDs model exhibits superior performance across all evaluated metrics, highlighting its efficacy in detecting early-stage breast cancer.

In terms of accuracy, FT-MDNNMDs reached a mean of 99.57% (95% CI: 99.21–99.83), showing high sensitivity and specificity across folds, and surpassing MDNNMDs by 4.22% and TL-MDNNMDs by 2.81%. The mean performance is reported across the 5 test folds together with 95% CI. This is computed according to Eq. (16).

16 $$\begin{aligned} CIs = \bar{x} \pm 1.96 \cdot \frac{s}{\sqrt{5}} \end{aligned}$$

where $\bar{x}$ is the mean across folds and *s* is the standard deviation. Moreover, to statistically validate the observed performance gains, a paired *t*-test is conducted on the fold-wise accuracy values obtained from the 5-fold cross-validation. For each fold, the accuracy of FT-MDNNMDs is paired with the accuracy of each baseline model, and a two-sided test was used with a significance level of $\alpha =0.05$. The results in Table 10 indicate that FT-MDNNMDs achieves statistically significant improvements over all baseline classifiers.

**Table 10:** Paired *t*-test p-values (Accuracy).

Comparison	p-value
FT-MDNNMDs vs. SVM	$p = 0.003$
FT-MDNNMDs vs. DT	$p = 0.002$
FT-MDNNMDs vs. NB	$p < 0.001$

### Per-class performance analysis

As shown in Table 11, the proposed FT-MDNNMDs model shows high performance across all classes. The Normal and Malignant categories achieve the highest precision and recall values, exceeding 99%. This reflects the strong discrimination between cancerous and non-cancerous cases. Slightly lower but still robust performance is observed for Stage II and Stage III classification, with F1-scores above 98%, indicating effective stage-level differentiation despite increased clinical complexity. The narrow standard deviations across folds further confirm the stability and generalization capability of the proposed framework.

**Table 11:** Per-class performance on the test folds (mean ± SD across 5 folds).

Class	Precision (%)	Recall (%)	F1-score (%)	AUC
Normal	$99.5 \pm 0.3$	$99.6 \pm 0.3$	$99.6 \pm 0.2$	$0.998 \pm 0.002$
Benign	$98.9 \pm 0.5$	$98.6 \pm 0.6$	$98.7 \pm 0.4$	$0.992 \pm 0.004$
Malignant	$99.2 \pm 0.4$	$99.4 \pm 0.4$	$99.3 \pm 0.3$	$0.996 \pm 0.003$
Stage II	$98.4 \pm 0.7$	$98.1 \pm 0.8$	$98.2 \pm 0.6$	$0.989 \pm 0.006$
Stage III	$98.6 \pm 0.7$	$98.9 \pm 0.6$	$98.7 \pm 0.5$	$0.991 \pm 0.005$

### Overall performance analysis

As shown in Table 12, the proposed FT-MDNNMDs model significantly outperforms traditional machine learning baselines evaluated on the same MDs dataset. Compared to classical methods, the improvement is substantial: FT-MDNNMDs surpasses SVM by 5.20%, DT by 5.99%, and NB by 8.14% in overall accuracy. This demonstrates the strong discriminative capability of the proposed framework in distinguishing between normal and cancerous cases. In terms of specificity, FT-MDNNMDs achieves 99.52%, outperforming MDNNMDs by 1.69%, TL-MDNNMDs by 2.01%, and NB by up to 10.18%, as reported in Table 12. Even when compared to the best-performing classical baseline (DT at 93.75%), the proposed model maintains a clear advantage of 5.77%. Similarly, recall reaches 99.63%, exceeding MDNNMDs by 5.04% and TL-MDNNMDs by 2.36%. This minimizes the risk of false negatives. The F1-score also attains 99.63%, reflecting a balanced trade-off between precision and recall. Precision further reaches 99.67%, reinforcing the ability of the model in positive case identification. Moreover, the Matthews Correlation Coefficient (MCC), a balanced metric suitable for imbalanced datasets, achieves 99.46%.

For a broader context, the proposed model is also compared with previously published breast cancer detection studies, as presented in Table 13. Across these external benchmarks, FT-MDNNMDs demonstrates improvements ranging from 3% to 10% in key performance metrics. It is important to note that this comparison is indicative, as prior studies are conducted on different datasets and experimental protocols. All results reported in Table 13 correspond to the final evaluation on the combined MDs dataset under the 5-fold cross-validation protocol.

**Table 12:** Performance comparison of proposed and baseline models on the combined MDs dataset (5-fold cross-validation).

Method	Accuracy (%)	Precision (%)	Recall (%)	F1-score (%)	Specificity (%)	MCC
SVM	94.46	95.64	95.56	95.60	98.91	94.49
NB	91.22	91.63	91.51	91.56	97.91	89.51
DT	93.54	93.98	93.95	93.95	98.49	92.43
MDNNMDs	95.37	95.61	95.56	95.58	98.90	94.50
TL-MDNNMDs	96.80	96.76	96.74	96.75	99.20	95.99
FT-MDNNMDs	99.57	99.67	99.63	99.57	99.89	99.46

**Table 13:** Indicative comparison with previously published breast cancer detection studies.

Study	Dataset used	Reported accuracy (%)
^59^	BreakHis	96.4
^60^	Mammography dataset	95.4
^61^	Multi-source dataset	94.6
^62^	Histopathology dataset	95.7
^63^	Molecular dataset	95.6
Proposed FT-MDNNMDs	Combined MDs dataset	99.57

To provide a balanced evaluation, the computational efficiency of the proposed FT-MDNNMDs model is compared with baseline machine learning classifiers under the same hardware configuration. The comparison considers approximate training time per fold, inference time per sample, and relative model complexity.

As shown in Table 14, classical machine learning models require minimal computational resources. The proposed FT-MDNNMDs model requires approximately 17.2 minutes per fold for training, 12.1 milliseconds per sample during inference, and a peak GPU memory usage of 4.6 GB. Although deep learning models require higher computational resources, the substantial gain in diagnostic accuracy justifies this trade-off in practical clinical settings.

**Table 14:** Computational efficiency comparison across models.

Model	Training time/fold (min)	Inference time (ms/sample)	Peak GPU memory (GB)
Naïve Bayes (NB)	0.8	0.4	0.1
Decision Tree (DT)	1.5	0.7	0.2
SVM (RBF)	3.9	2.1	0.3
MDNNMDs	19.6	13.4	4.8
TL-MDNNMDs	15.8	11.7	4.5
FT-MDNNMDs	17.2	12.1	4.6

## Limitations and deployment challenges

The limitations of the proposed research include: the private dataset was collected from a single healthcare institution within one geographical region, which may limit the generalisability of the model to other populations, clinical environments, and imaging equipment. Multi-centre validation across diverse demographic and imaging settings is required to further assess robustness. Moreover, while multiple imaging modalities were incorporated, certain modalities, such as thermography are not part of standard breast cancer screening protocols in many clinical settings. Therefore, the practical deployment of the full multimodal framework may depend on local infrastructure availability. Furthermore, no external independent test set or prospective validation study is conducted. Future work should include external dataset validation and real-world prospective evaluation to further establish clinical reliability. One more limitation is that Stage I cases are not included in this research due to limited availability within the collected dataset; therefore, stage-level classification in this study is restricted to Stage II and Stage III cases. Integration with hospital information systems and imaging workflows requires interoperability with existing PACS and electronic health record (EHR) systems. Additionally, reliable deployment demands adequate computational infrastructure, especially in resource-constrained settings. Data privacy, cybersecurity compliance, and adherence to regulatory standards are critical factors that must be addressed before clinical adoption.

## Conclusion

The proposed research addresses the challenge of breast cancer detection and emphasizes the need for enhanced accuracy in early-stage diagnosis. The proposed MDNNMDs take advantage of TCGA and a private dataset from Pakistani hospitals, which shows their efficiency in the detection of both tumor and non-tumor breast cancer conditions. Through meticulous preprocessing techniques, including image filtering, data handling, and principal component analysis (PCA), the model demonstrates improved image quality and feature relevance. The application of transfer learning and fine-tuning further enhances the accuracy of the proposed model, particularly in identifying early stage II and stage III breast cancer and effectively distinguishing between benign and malignant cases. Comparative analysis with traditional machine learning algorithms such as SVM, DT, and NB reveals the superior performance of the proposed FT-MDNNMDs, achieving an impressive accuracy of 99.57%. Notably, the model excels in terms of MCC, F-score, and recall, achieving scores of 99.46%, 99.57%, and 99.63%, respectively. Beyond algorithmic comparisons, the research positions the proposed model against existing breast cancer detection models, unveiling its superiority with a 1. 5% to 3. 0% increase in precision. The results show the potential of the FT-MDNNMDs framework for significantly improving breast cancer detection accuracy.

Future work should include external dataset validation and real-world prospective evaluation to further establish clinical reliability. Moreover, in the future, we are aiming to focus on integrating advanced imaging techniques such as functional MRI or molecular imaging, and ensemble learning approaches could also be explored to improve the robustness of models, and real-time monitoring with predictive analytics may enable the proposed FT-MDNNMDs to more accurately identify early patterns indicative of breast cancer tumor. Additionally, there is potential for developing models tailored to individual patient profiles for personalized medicine, and the incorporation of multi-omics data could provide a comprehensive molecular understanding. Further investigations into human-computer interaction in clinical settings and the societal implications of implementing advanced detection models will contribute to the continuous improvement of the accuracy of breast cancer diagnosis. Also, model optimization for lightweight deployment will be considered

## Data Availability

The private clinical dataset used in this study cannot be publicly shared due to ethical restrictions. Access to de-identified data may be considered upon reasonable request and subject to approval by the Institutional Review Board. Publicly available datasets used in the transfer-learning stage (e.g., BreakHis and INbreast) are accessible through their respective repositories.
